# People Living With Dementia Living Independently: The Role of Older Americans Act Services

**DOI:** 10.1093/geroni/igaf122.883

**Published:** 2025-12-31

**Authors:** Ian Matthew Nelson, Heather Menne, Kate Singer, Emerson McSparran

**Affiliations:** Miami University, Oxford, Ohio, United States; Miami University, Oxford, Ohio, United States; Miami University, Oxford, Ohio, United States; Miami University, Oxford, Ohio, United States

## Abstract

Older Americans Act (OAA) funding provides community-based services to help individuals age 60 and over live independently in their home and communities. The National Survey of Older Americans Act Participants (NSOAAP) is a nationally representative survey of OAA program participants receiving services such as case management, homemaking, home-delivered meals, transportation, and congregate meals. We used the 2023 NSOAAP survey to examine whether the receipt of OAA services helps participants living with dementia (PLWD) remain independent and at home. The percentage of PLWD responding to the survey across the 5 programs varied between 13% home delivered meal services and 4% for those attending congregate meals. Very high percentages report that these services help them live independently regardless of dementia status (75-98%). Fewer PLWD report that transportation services assist them with remaining at home when compared to those without dementia (86% versus 93%). Finally, a significantly higher percentage of PLWD report that congregate meals help them remain home when compare to those without dementia (91% versus 75%). A smaller portion of the sample, up to 7% of OAA participants, are people living alone with dementia (PLAWD). Eighty-one to 100% of PLAWD report that the service helps them live independently. Based on these results of vulnerable OAA participants, many opportunities are available for further research on other factors contributing to participants’ perceptions and ability to remain independently. In addition, policymakers can use these results to explore mechanisms that enhance supports for older adults living with dementia to live independently.

